# Supplementary material for: Heterochronic developmental shifts underlie floral diversity within Jaltomata (Solanaceae)
Source: EvoDevo. 2017 Oct 23;8:17. doi: 10.1186/s13227-017-0080-z (PMC5651583; doi:10.1186/s13227-017-0080-z)
Supplement: Supplementary file 2 — Additional file 2: Figure S1. Measured morphological traits on mature flowers. Floral organs were measured on three flowers per individual, for at least three individuals per species, using hand-held digital calipers to the nearest 0.01 mm. Calyx diameter was measured as the widest planar distance across the calyx (outer most whorl); sepal length from the tip of the sepal to the center of the calyx; corolla diameter as the widest planar distance across the corolla (second whorl); corolla depth as the vertical distance from the base of the corolla to its highest point; petal length from the tip of the petal to the center of the corolla base; corolla fusion from the base of the corolla to the highest point of congenitally fused corolla; petal lobe length from the lowest point of non-fused petal edge to petal tip; stamen length from the base of the stamen (anther filament) to the tip of the anther; ovary diameter as the widest planar distance across the base of the ovary (inner most whorl); and style length from the base of the style to its tip (stigma). [file 13227_2017_80_MOESM2_ESM.pdf]

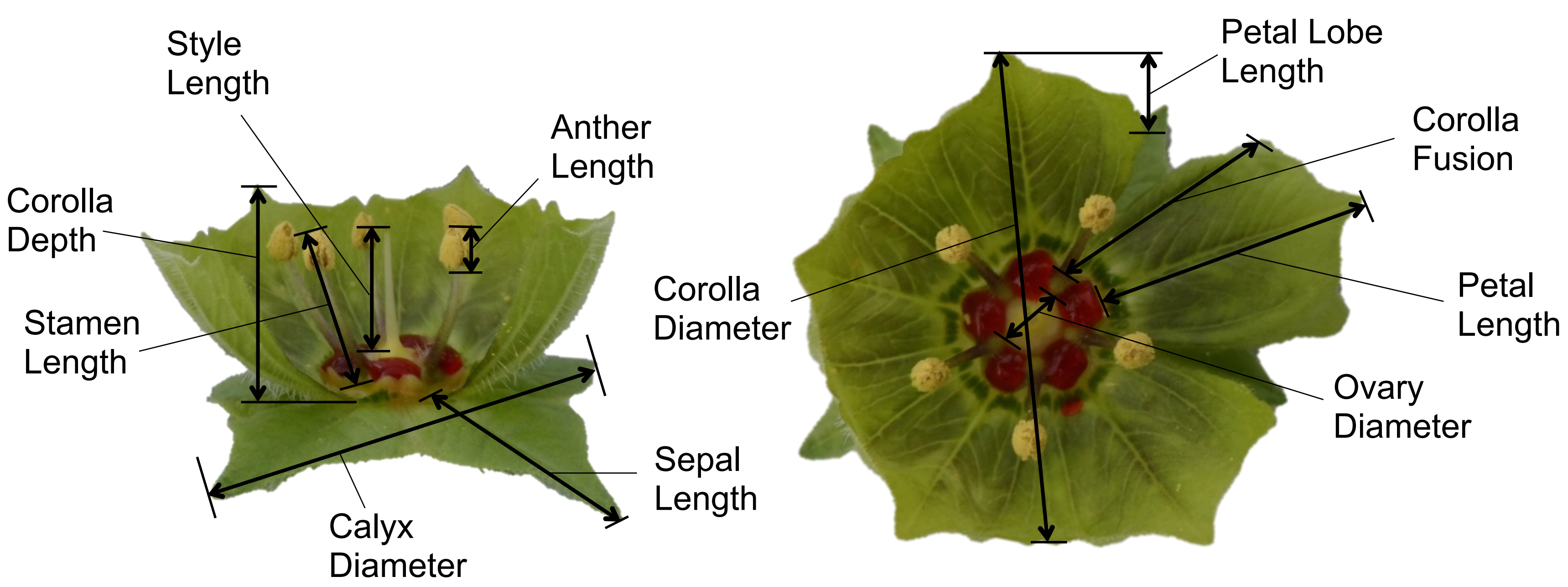

Figure S1. Measured morphological traits on mature flowers. Floral organs were measured on three flowers per individual, for at least three individuals per species, using hand-held digital calipers to the nearest 0.01 mm. Calyx diameter was measured as the widest planar distance across the calyx (outer most whorl); sepal length from the tip of the sepal to the center of the calyx; corolla diameter as the widest planar distance across the corolla (second whorl); corolla depth as the vertical distance from the base of the corolla to its highest point, petal length from the tip of the petal to the center of the corolla base; corolla fusion from the base of the corolla to the highest point of congenitally fused corolla; petal lobe length from the lowest point of non-fused petal edge to petal tip; stamen length from the base of the stamen (anther filament) to the tip of the anther; ovary diameter as the widest planar distance across the base of the ovary (inner most whorl); and style length from the base of the style to its tip (stigma).
